# Evaluation of an automated von Willebrand factor glycoprotein IbM activity assay compared with 3 alternative von Willebrand factor activity assays

**DOI:** 10.1016/j.rpth.2024.102422

**Published:** 2024-04-26

**Authors:** Kenneth D. Friedman, Martina Böhm-Weigert, Nicole DeSimone, Dennis J. Dietzen, Charles Eby, Cynthia Flickinger, Walter Hoyer, Mareike Kahl, Kandice Kottke-Marchant, Thomas L. Ortel, Jürgen Patzke, Steven W. Pipe, Morgan Stuart, Ayse Anil Timur, Ravindra Sarode

**Affiliations:** 1 Medical Science Institute, Versiti Blood Center of Wisconsin, Milwaukee, Wisconsin, USA; 2 Department of Medical Affairs, Siemens Healthcare Diagnostics Products GmbH, Marburg, Germany; 3 Pathology and Internal Medicine (Hematology/Oncology), University of Texas Southwestern Medical Center, Dallas, Texas, USA; 4 Department of Pathology and Immunology, Washington University, St. Louis, Missouri, USA; 5 Department of Clinical Evaluation, Siemens Healthcare Diagnostics Inc, Glasgow, Delaware, USA; 6 Pathology and Laboratory Medicine Institute, Cleveland Clinic Foundation, Cleveland, Ohio, USA; 7 Department of Medicine, Duke University, Durham, North Carolina, USA; 8 Department of Assay Development, Siemens Healthcare Diagnostics Products GmbH, Marburg, Germany; 9 Departments of Pediatrics and Pathology, University of Michigan, Ann Arbor, Michigan, USA

**Keywords:** diagnosis, reference range, reproducibility of results, ristocetin cofactor, von Willebrand disease, von Willebrand factor

## Abstract

**Background:**

To overcome deficiencies of the traditional von Willebrand factor (VWF) ristocetin cofactor activity assay (VWF:RCo), several automated assays for VWF platelet-binding activity have been developed. Information on the performance of these assays and their diagnostic utility remains limited.

**Objectives:**

To validate the VWF:glycoprotein IbM assay INNOVANCE VWF Ac and compare it with an automated VWF:RCo assay as well as with an automated assay and a manual VWF:Ab assay and to generate reference ranges and analyze reproducibility of the VWF:glycoprotein IbM assay.

**Methods:**

Clinical sites enrolled healthy subjects and patients representing the intended use population; VWF activity assays were performed, and results were analyzed. The performance of the INNOVANCE VWF Ac assay was also compared between the BCS XP System and the CS-2500 and CS-5100 analyzers.

**Results:**

The INNOVANCE VWF Ac assay correlated well with the VWF:RCo assay and the automated HemosIL VWF:Ab assay, with Pearson coefficients of >.9 and a predicted bias of ≤5.0 IU/dL at VWF levels of 30 IU/dL and ≤5.8 IU/dL at the levels of 50 IU/dL, but correlation and bias were not as good when compared with the REAADS manual VWF:Ab assay. Reference ranges observed for healthy subjects correlated well with previously published findings. Reproducibility of the INNOVANCE VWF Ac assay on the BCS XP System and the CS analyzers was excellent, as was correlation among devices.

**Conclusion:**

The characteristics of the INNOVANCE VWF Ac assay regarding comparability with other VWF activity assays, reference ranges, and precision support the use of this assay for evaluation of patients with concern for von Willebrand disease.

## Essentials

• Measurement of von Willebrand factor (VWF) activity is important to diagnose von Willebrand disease.
• A new assay to measure VWF activity without ristocetin was validated in the US population.
• The assay compares well with other commercial VWF activity assays available in the United States.
• Reference ranges were defined, and precision data demonstrated the good performance of the assay.

## 1 Introduction

von Willebrand disease (VWD) is the most common inherited bleeding disorder [[1], [2], [3]] attributed to either quantitative or qualitative defects of von Willebrand factor (VWF), a multimeric plasma glycoprotein (GP) [2]. VWF possesses multiple functions to support hemostasis, including binding to subendothelial matrix components (most notably collagen), binding to platelets via GPIb to support platelet adhesion at sites of vascular injury, and acting as a chaperone for factor (F)VIII in circulation [2]. Shear forces are responsible for both physiological activation of VWF to result in platelet interactions as well as control of VWF multimers through cleavage by ADAMTS-13 [4,5]. The complexity of VWF structure, function, and regulation results in multiple forms of VWD.

The International Society on Thrombosis and Haemostasis (ISTH) recognizes 6 different types of inherited VWD variants [6]. Type 1 is defined by quantitative VWF deficiency. Type 1C, characterized by increased clearance of VWF, may represent as many as 15% of individuals with type 1 disease [7]. Complete absence of VWF is termed type 3 VWD. Type 2 VWD represents a heterogeneous set of variants characterized by qualitative defects of VWF, including type 2A (absence of high-molecular-weight multimers), type 2B (enhanced interaction of VWF with platelets, increasing the clearance of high-molecular-weight VWF multimers and platelets), type 2M (dysfunction of VWF interaction with either platelets or collagen that is not attributable to loss of high-molecular-weight multimers), and type 2N (impaired interaction of the VWF with coagulation FVIII). Along with these recognized inherited forms of VWD, additional forms include inheritable platelet-type VWD, attributable to gain-of-function mutations in platelet GPIb [8], and acquired von Willebrand syndrome (AVWS), which may occur as a consequence of multiple pathologic mechanisms [2,9].

A number of laboratory studies are required for the diagnosis of VWD and its further differentiation into subtypes [4,5]. It is recommended by the ASH ISTH NHF WFH guideline (American Society of Hematology, International Society on Thombosis and Haemostasis, National Hemophilia Foundation, and World Federation of Hemophilia, respecitvely) [1] and the National Heart, Lung, and Blood Institute (NHLBI) guideline [10] that the initial laboratory assessment should include 3 assays: VWF antigen, a measure of VWF platelet-binding function, and FVIII coagulant activity. The traditional assay for VWF platelet-binding function is the VWF ristocetin cofactor activity assay (VWF:RCo) [11]. This assay was developed as a platelet agglutination method in the 1970s using stabilized platelets and the antibiotic ristocetin [12]. Ristocetin induces VWF binding to platelet GPIb receptors, resulting in agglutination of platelets and a decrease in turbidity. The main drawbacks of VWF:RCo by aggregometry were its very high imprecision and the lower limit of its measuring interval, typically 10 to 20 IU/dL [13]. Automation of the VWF:RCo assay allowed improved assay precision, but the limit of detection remains an issue [11,13]. Possible technical solutions to improve the limit of detection were published (reviewed by Just [14] in 2017), but such assay modifications are not generally available through clinical laboratories. Also, a common variant (especially in the population of African descent) within the VWF gene encoding for incorporation of aspartic acid rather than histidine at amino acid position 1474 (p.D1472H) affects the sensitivity of VWF to ristocetin-based activation, leading to artificially low assay results [15].

A number of alternative assays involving microbeads have been developed to satisfy the demand for assay automation and improved precision, with nomenclature designated by the VWF Subcommittee of the Standardization and Scientific Committee of the ISTH [11]. A monoclonal antibody (mAb) binding-based assay for VWF activity (nomenclature abbreviation VWF:Ab) was originally formulated as an enzyme-linked immunosorbent assay (ELISA) using mAb directed against a VWF epitope involved in VWF-GPIb binding (eg, REAADS VWF activity assay from Corgenix Medical Corp) [16,17]. This methodology was subsequently formulated into an automated latex immunoassay (HemosIL VWF activity from Werfen), with the automated method performing better than the ELISA method and showing good correlation with the VWF:RCo assay [18,19]. However, because this assay reports binding of a mAb to VWF rather than VWF to GPIb, some questions remain as to what extent this assay fully reports VWF platelet-binding function [11]. Another assay measures ristocetin-triggered GPIb binding of VWF to a recombinant GPIb fragment adhered to either latex or magnetic microparticles (nomenclature VWF:GPIbR, commercially available assays from Werfen) [20,21]. Finally, an automated assay using gain-of-function mutants of GPIb (nomenclature VWF:GPIbM) allows measurement of the interaction of VWF platelet-binding activity in the absence of ristocetin (INNOVANCE VWF Ac assay from Siemens Healthineers) [22].

As preliminary work to determine whether the diagnostic accuracy of the automated VWF:GPIbM assay was equivalent or potentially superior to the standard-of-care assays for VWD in a cohort of US patients, assay precision and reference ranges were established for the INNOVANCE VWF Ac assay on the BCS XP System (Siemens Healthineers) and the CS-2500 and the CS-5100 automated analyzers (both from Sysmex Corp). In addition, we analyzed the correlation between the INNOVANCE VWF Ac assay and an automated VWF:RCo assay and also compared these results with 2 VWF:Ab assays.

## 2 Methods

### 2.1 Overview of study design

**Figure 1:**
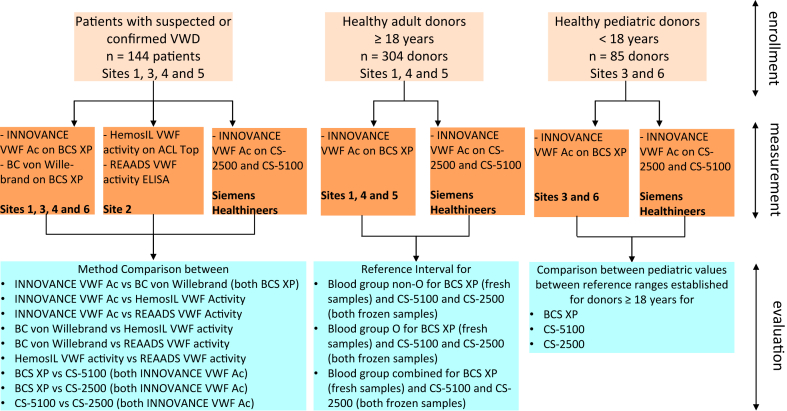
Overview of method comparison, reference range, and healthy minor study components. Site 1 is Cleveland Clinic Foundation, site 2 is Duke University, site 3 is University of Michigan, site 4 is University of Texas Southwestern Medical Center, site 5 is Versiti Blood Center of Wisconsin, and site 6 is Washington University. VWD, von Willebrand disease; VWF, von Willebrand factor.

The study was performed at 6 clinical sites in the United States: (1) Cleveland Clinic Foundation (Cleveland, Ohio), (2) Duke University (Durham, North Carolina), (3) University of Michigan (Ann Arbor, Michigan), (4) University of Texas Southwestern Medical Center (Dallas, Texas), (5) Versiti Blood Center of Wisconsin (Milwaukee, Wisconsin), and (6) Washington University (St. Louis, Missouri) and in a laboratory owned by Siemens Healthineers. The institutional review boards of each site enrolling subjects approved this study. Enrolled subjects signed informed consent to participate. One site used residual samples for which informed consent was waived. The study consisted of 4 different components: method comparison, reference range, healthy minors (see Figure 1 for an overview of these 3 components), and reproducibility.

### 2.2 Method comparison

For the method comparison study, patients were enrolled at 4 clinical sites (Figure 1). The inclusion criterion was a suspected or confirmed VWF disorder (VWD or AVWS). Additionally, sites included some samples from persons with hemophilia and platelet dysfunction as well as some samples from patients with suspected elevated VWF levels to cover the high end of the measuring interval. Clinical diagnosis was based on the diagnostic criteria used at subject-enrolling sites or referring institutions. A blood sample was obtained from each enrolled subject. The sites measured VWF activity with the INNOVANCE VWF Ac assay and BC von Willebrand Reagent on the BCS XP System (reagents and system from Siemens Healthineers) using either fresh samples (within 4 hours of drawing blood) or after frozen storage at ≤−70 °C (for a maximum of 1 month). Previous studies (not described here) demonstrated that the INNOVANCE VWF Ac assay results do not significantly differ between fresh and frozen samples. The subject-enrolling sites sent frozen sample aliquots to site 2 and to Siemens Healthineers. Site 2 measured VWF activity with the HemosIL VWF activity assay on the ACL TOP system (reagent and analyzer from Werfen) and with the REAADS VWF activity test kit from Corgenix Inc. Siemens Healthineers laboratory measured VWF activity (with the INNOVANCE VWF Ac assay on the CS-2500 and CS-5100 analyzers; both from Sysmex Corp). Assays were performed following the manufacturer’s recommended procedures, and only data within the reportable range suggested by the assay manufacturer were statistically analyzed. Assay name, manufacturer name, ISTH nomenclature, reportable intervals, and calibrators are listed in Supplementary Table S1.

### 2.3 Reference range

For the determination of the reference ranges, healthy adults (≥18 years of age) were enrolled at 3 clinical sites (Figure 1). Blood group was determined by standard transfusion medicine practices. Reference ranges were established for blood group O and blood group non-O with a minimum of 150 healthy donors, respectively. Donor recruitment was targeted to reflect the ethnic distribution of the US population and to be balanced regarding sex and age. Each site used a different INNOVANCE VWF Ac reagent/calibrator lot combination and was required to enroll at least 40 donors per subgroup. Blood samples were measured on the BCS XP System within 4 hours of drawing blood at subject-enrolling sites and on the CS-2500 and CS-5100 analyzers within 3 months of frozen storage (≤−70 °C) at Siemens Healthineers.

### 2.4 Healthy minors

In addition to the reference range study performed for adults (*≥*18 years of age), VWF activity using the INNOVANCE VWF Ac assay was measured in healthy minors (>4 weeks and <18 years of age), as outlined in Figure 1. Healthy minors admitted for routine checkup or minor elective surgery were enrolled at 2 clinical sites (Figure 1). Blood samples from each enrolled subject were measured on the BCS XP System (at enrolling sites) as well as on the CS instruments (at Siemens Healthineers).

### 2.5 Reproducibility/precision

The reproducibility of the INNOVANCE VWF Ac assay on the BCS XP System was investigated by measuring control plasma N and control plasma P (both from Siemens Healthineers) and 4 plasma pools (2 of which were diluted with VWF-deficient plasma and 1 of which spiked with Humate-P concentrate from CSL Behring). The sample pools were prepared by Siemens Healthineers to cover the measuring interval of the INNOVANCE VWF Ac assay (4%-300% of normal level, which equals 4-300 IU/dL). The pools were aliquoted and sent frozen to the 3 participating clinical sites (sites 1, 4, and 5). The sites measured reproducibility samples on 5 days, with 2 runs per day and 3 replicates of each sample per run on the BCS XP System using the same reagent/calibrator lot combination. Siemens Healthineers investigated the variability between reagent lots (*n* = 3) by measuring controls and plasma pools on 20 days, with 2 runs per day and 2 replicates per run on 1 BCS XP System. Two reagent lots were used in combination with 1 calibrator lot, and the third reagent lot was used in combination with 3 calibrator lots. Siemens Healthineers further investigated the precision of the INNOVANCE VWF Ac assay on the CS-2500 and CS-5100 analyzers by measuring controls and pools with 1 reagent/calibrator lot on 20 days, with 2 runs per day and 2 replicates per run on 1 instrument as well as on 3 different instruments (5 days, 2 runs per day, 4 replicates per run).

### 2.6 Statistical analysis

The comparability between the INNOVANCE VWF Ac assay and other VWF activity assays was assessed by Passing–Bablok regression analysis and by Bland–Altman analysis, as described in CLSI document EP09c [23] using JMP 17.0.0 software on a Windows 10 system.

The reference ranges were calculated nonparametrically (as described in CLSI document EP28-A3c) [24] using SAS 9.4 software (SAS Institute) on a Windows 10 system as 2-sided 95% CIs (2.5th/97.5th percentiles).

The results from healthy minors were statistically compared with the results of the adult reference population by a 2-sample *t*-test (with unequal variances) by using log-transformed data. Since even after log transformation, some data sets showed deviations from normality, all results and conclusions were confirmed by a nonparametric Wilcoxon test. All calculations were performed with JMP 17.0 software (JMP Statistical Discovery LLC) on a Windows 10 system. In all statistical tests, a *P* value of <.05 was considered to indicate a statistical significance. Individual results of >300 IU/mL occurring once in each population (adult/healthy minors) in the non-O blood groups were set to 300 for statistical analysis and plotting.

The reproducibility and precision data were evaluated according to CLSI document EP05-A3 [25]. The reproducibility data were analyzed by a 3-factorial, fully nested analysis of variance with the factors site, day (nested within site), and run (nested within day and site) using validated internal software on a Windows 10 system. The precision data were also analyzed by a 3-factorial, fully nested analysis of variance with the factor’s reagent lot, day (nested within site), and run (nested within day and site) for each sample.

## 3 Results

### 3.1 Method comparison

For the method comparison study, 131 patients were included at 4 different clinical sites, and 13 samples were purchased from a commercial vendor. An overview of study population characteristics is given in Table 1, showing inclusion of 46 pediatric patients (32%) and 98 adult patients (68%). Fifty-nine patients (42%) had been previously diagnosed with VWD, as detailed in Table 1. Four patients with a diagnosis of AVWS and 69 patients with a clinical suspicion of VWD or AVWS were included. The population represents the ethnicity/race distribution of the US population.

**Table 1:** Characteristics of the study population.

Characteristic	*n* (%) of subjects Method comparison	*n* (%) of subjects Reference range	*n* (%) of subjects Healthy minors
Age (y)			
<18	46 (32)	—	85 (100)
≥18	98 (68)	304 (100)	—
Ethnicity/race			
White, not Hispanic or Latino (%)	120 (83)	199 (65)	58 (68)
White, Hispanic or Latino (%)	6 (4)	25 (8)	4 (5)
Black	14 (10)	42 (14)	21 (25)
Asian or others	4 (3)	38 (13)	2 (2)
Sex			
Female	103 (72)	156 (51)	45
Male	41 (28)	148 (49)	40
Previously diagnosed with VWD			
Type 1	29	—	—
Type 2A/2B/2M/2N	7/5/4/3	—	—
Type 3	8	—	—
Type not defined	3	—	—
Patients with AVWS	4	—	—
Suspected for VWD	69	—	—
Other conditions			
Hemophilia A	5	—	—
Platelet dysfunction	1	—	—
Suspected elevated levels of VWF	3	—	—
Diluted samples from healthy donors	3	—	—
Healthy subjects	—	304 (100)	85 (100)

AVWS, acquired von Willebrand syndrome (diagnosis as recorded in the medical file); VWD, von Willebrand disease; VWF, von Willebrand factor.

**Figure 2:**
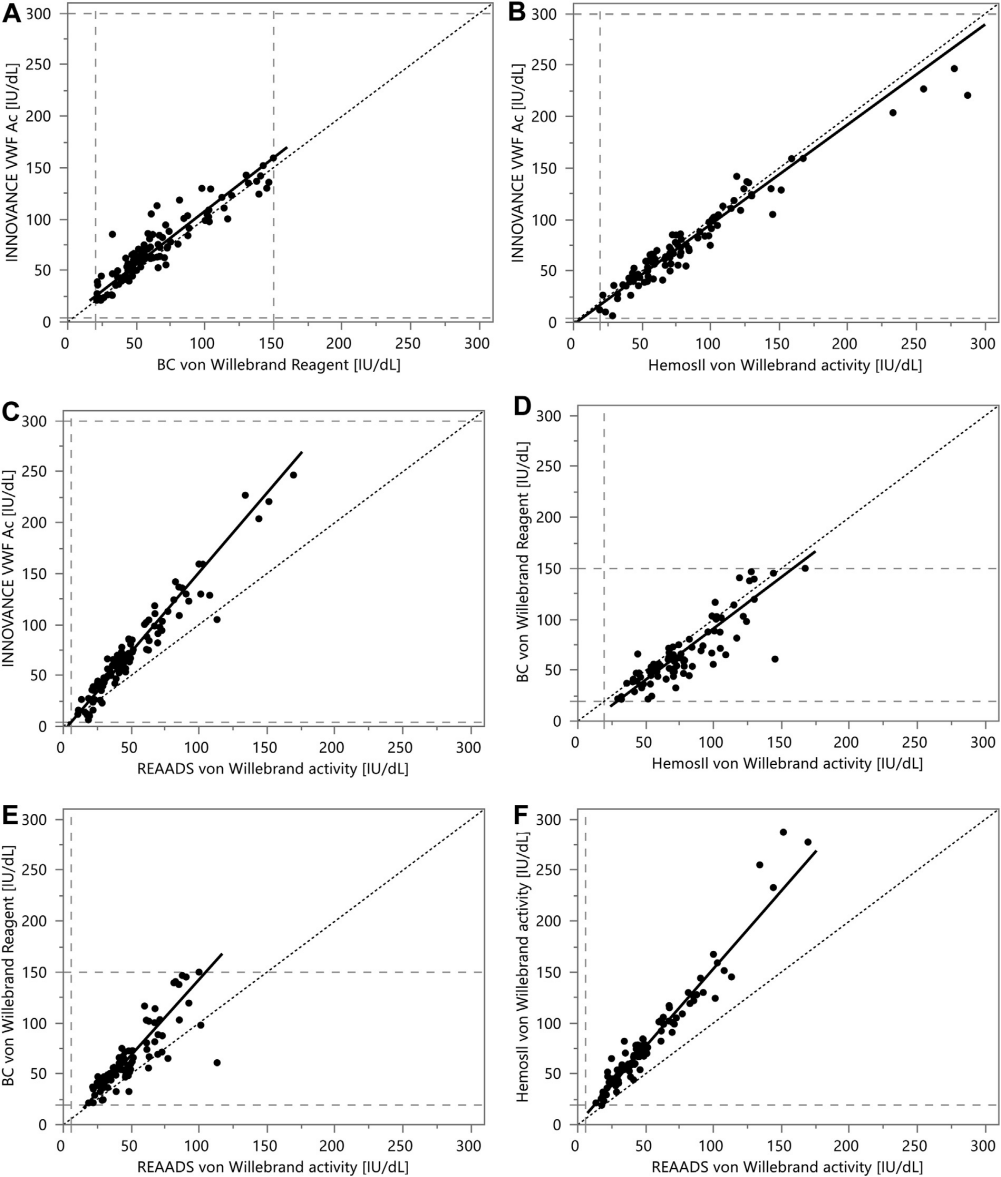
Passing–Bablok regression analysis for all reagent combinations. (A–C) INNOVANCE VWF Ac assay on BCS XP System vs (A) BC von Willebrand Reagent on BCS XP System, vs (B) HemosIL VWF activity assay on ACL TOP system, and vs (C) REAADS VWF activity ELISA assay. (D–E) BC Von Willebrand Reagent on BCS XP System vs (D) HemosIL VWF activity assay on ACL TOP system, and vs (E) REAADS VWF activity ELISA assay. (F) HemosIL VWF activity assay on ACL TOP system vs REAADS VWF activity ELISA assay. The dashed lines in the plots show the corresponding measuring intervals of the methods. The lines of identity are dotted. The results for intercept, slope, predicted bias at 30 IU/dL and 50 IU/dL, and correlation coefficient are listed in Table 2. The corresponding Bland–Altmann plots are shown in Supplementary Figure S1. ELISA, enzyme-linked immunosorbent assay; VWF, von Willebrand factor.

The dataset of the method comparison study was evaluated by Passing–Bablok regression and Bland–Altmann analysis. Samples not meeting the sample quality criteria for the study (Supplementary Table S2) were excluded. Regression analysis is summarized in Table 2 and Figure 2. The INNOVANCE VWF Ac assay (the automated VWF:GPIbM assay) correlated well with the BC von Willebrand Reagent (the automated VWF:RCo assay) and with the HemosIL VWF activity assay (the automated VWF:Ab assay), with Pearson correlation coefficients of >.9 and a predicted bias at 30 and 50 IU/dL of 5.0 and 5.8 IU/dL compared with the VWF:RCo assay and −2.8 and −3.4 IU/dL compared with the automated VWF:Ab assay. The REAADS VWF activity assay (the manual VWF:Ab assay) compared less well with the other VWF activity assays, with high values for the slope (≥1.5) resulting in high values for the predicted bias at 30 IU/dL (between 8.7 and 16.4 IU/dL) and 50 IU/dL (between 18.5 and 27.2 IU/dL). The Bland–Altman plots provided in Supplementary Figure S1 demonstrate a small mean difference among the automated assays and a large mean difference when comparing the manual VWF:Ab assay with the other assays. To identify outliers, residuals of the regression were computed for all 6 comparisons and investigated in normal-quantile and outlier box plots. Five samples were identified as potential outliers in at least 1 of the 6 comparisons (Supplementary Table S3). Two samples had high VWF activity results (>100 IU/dL for all methods), and discrepancies may be thus clinically less relevant. The discrepancies in the other samples may be of clinical relevance since they were found in the range of <100 IU/dL. All of these samples had considerably lower VWF activity with the VWF:RCo method compared with the VWF:GPIBM and the automated VWF:Ab methods.

**Table 2:** Method comparison between reagents analyzed by Passing–Bablok regression.

Method comparison	No. of samples^a^	No. of samples included in regression analysis^b^	Slope	Intercept (IU/dL)	Pearson correlation coefficient	Predicted bias at 30 IU/dL (IU/dL)	Predicted bias at 50 IU/dL (IU/dL)
INNOVANCE VWF Ac on BCS XP vs							
BC von Willebrand on BCS XP	140	104	1.04	3.9	.927	5.0	5.8
HemosIL VWF activity on ACL TOP	120	100	0.97	−2.0	.971	−2.8	−3.4
REAADS VWF activity ELISA	120	107	1.56	−4.9	.965	11.9	23.1
BC Von Willebrand on BCS XP vs							
HemosIL VWF activity ACL TOP	116	86	1.01	−9.7	.861	−9.5	−9.3
REAADS VWF activity ELISA	116	86	1.49	−5.8	.832	8.7	18.5
HemosIL VWF activity on ACL TOP vs							
REAADS VWF activity ELISA	119	100	1.53	0.53	.970	16.4	27.0

ELISA, enzyme-linked immunosorbent assay; VWF, von Willebrand factor.

a From the total cohort (*N* = 144), some samples needed to be excluded from the method comparison study because of pre- or postanalytical exclusion criteria (see Supplementary Table S2 for details).

b Patients with values outside of the measuring interval with one or both reagents had to be excluded from the statistical regression analysis and are listed in Supplementary Table S3.

Samples excluded from the statistical analysis of the method comparisons (*n* = 38) due to VWF activity results outside of the measuring intervals are listed in Supplementary Table S4. The results demonstrate good comparability between reagents, except for 3 samples (sample numbers 20, 21, and 24 in Supplementary Table S4). Two samples (both from patients previously diagnosed with type 2B VWD) demonstrated considerably lower values with the VWF:GPIbM assay (<4 and 6.4 IU/dL), whereas both VWF:Ab assays showed higher values between 17.3 and 28.5 IU/dL with the VWF:Ab methods. The VWF:RCo assay reported <20 IU/dL for both samples. The third sample with considerable differences between reagents demonstrated a result of <20 IU/dL with VWF:RCo assay, whereas all other methods reported values of >40% IU/dL. The reason for these discrepancies was not investigated.

The VWF:GPIbM assay did not generate a valid result in 2 samples (1 from a patient with AVWS and 1 from a patient with VWD type 1C); measurement in the normal setting demonstrated a result of <20 IU/dL, prompting remeasurement in the low setting that showed a result of >25 IU/dL. The reason for this discrepancy is not known. As expected, the number of results outside the measuring interval was highest for BC von Willebrand Reagent and lowest for the INNOVANCE VWF Ac assay.

**Figure 3:**
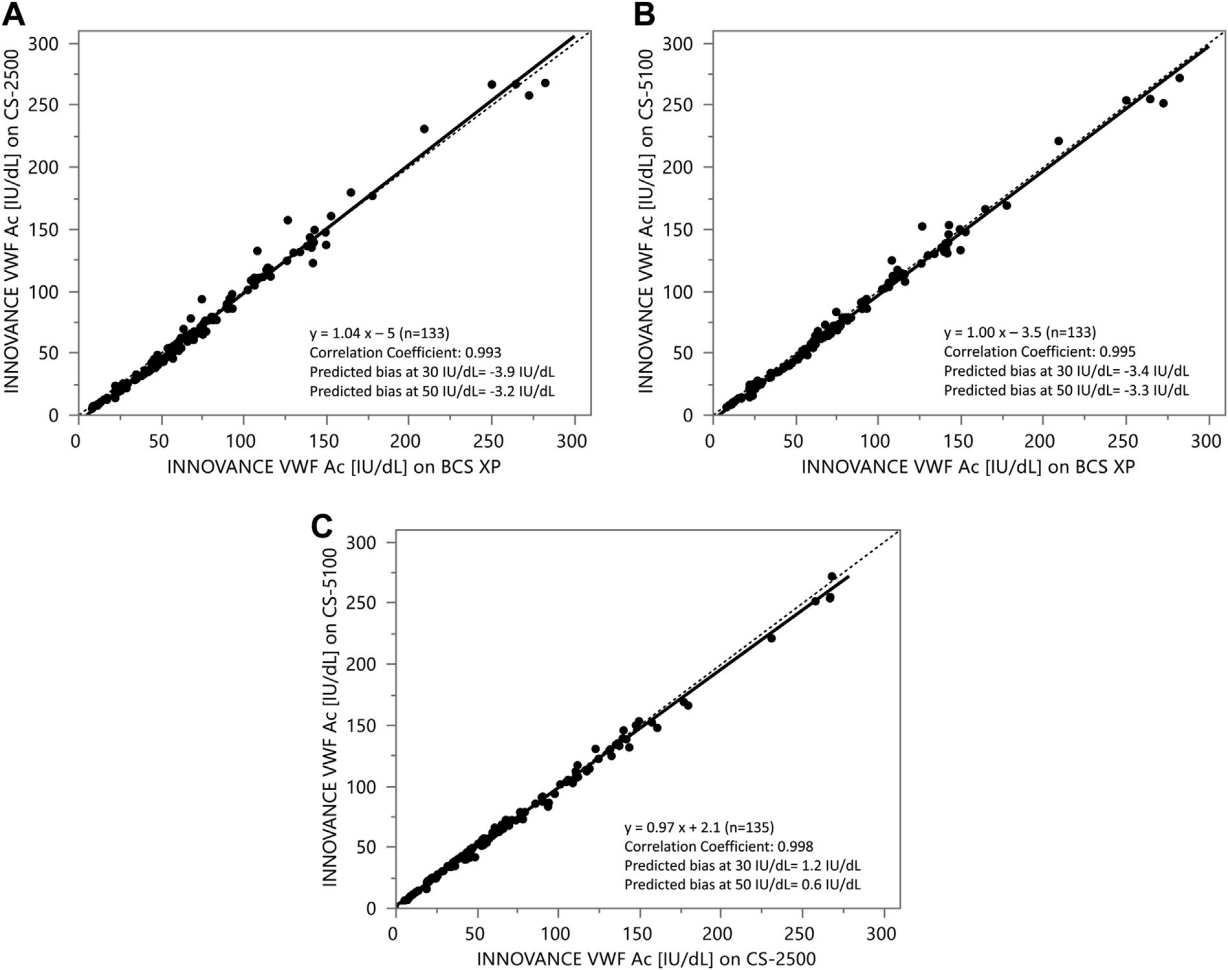
Passing–Bablok regression analysis for the INNOVANCE VWF Ac assay between different analyzers. (A) CS-2500 vs BCS XP, (B) CS-5100 vs BCS XP, and (C) CS-5100 vs CS-2500. From the total cohort (*N* = 144), 2 samples had no valid result for the CS-5100 analyzer and were thus excluded. Samples with results outside the measuring interval also needed to be excluded from statistical evaluation. VWF, von Willebrand factor.

The method comparison between analyzers (BCS XP System, CS-5100, and CS-2500) performed for the VWF:GPIbM assay demonstrated that the analyzers compare very well (Figure 3) with correlation coefficients of ≥.993, slopes of 0.97 to 1.04, and predicted biases of ≤3.9 IU/dL.

### 3.2 Reference ranges

We included 304 apparently healthy donors representing the US race and ethnic distribution at 3 different sites (Table 1). The population was balanced regarding gender, age (26% between 18 and 30 years, 35% between 31 and 50 years, 29% between 51 and 65 years, and 10% over 65 years), and ABO blood group (*n* = 150 with blood group O and *n* = 152 with non-O blood groups). The reference ranges presented in Table 3, calculated as 2-sided 95% CIs (2.5th/97.5th percentiles), demonstrate lower values for blood group O compared with non-O blood groups. The 2.5th percentiles for all blood groups combined ranged between 47.1 and 50.7 IU/dL, and the 97.5th percentiles ranged between 197.8 and 208.2 IU/dL. The reference ranges compared very well among the different analyzers.

**Table 3:** Reference ranges for the INNOVANCE VWF Ac assay on BCS XP and CS-2500 and CS-5100 systems.

Analyzer	No. of donors^a^	Minimum (IU/dL)	2.5th percentile (IU/dL)	Median	97.5th percentile (IU/dL)	Maximum (IU/dL)
BCS XP						
ABO blood groups combined	302	42.9	50.7	110.5	203.5	>300.0
Blood group O	150	42.9	49.0	90.6	178.9	196.9
Blood group non-O	152	49.5	60.7	131.9	214.1	>300.0
CS-2500						
ABO blood groups combined	303	38.4	48.5	109.3	208.2	>300.0
Blood group O	150	38.4	43.2	89.6	182.5	206.5
Blood group non-O	153	42.2	54.0	130.1	215.9	>300.0
CS-5100						
ABO blood groups combined	303	38.0	47.1	109.1	197.8	290.1
Blood group O	150	38.0	42.3	87.5	175.6	187.3
Blood group non-O	153	41.5	54.0	129.2	201.7	290.1

a From the total cohort (*N* = 304), 2 samples were excluded on the BCS XP because storage time was exceeded. On the CS instruments, 1 sample needed to be excluded because sample was not shipped to the Siemens Site.

### 3.3 Healthy minors

**Figure 4:**
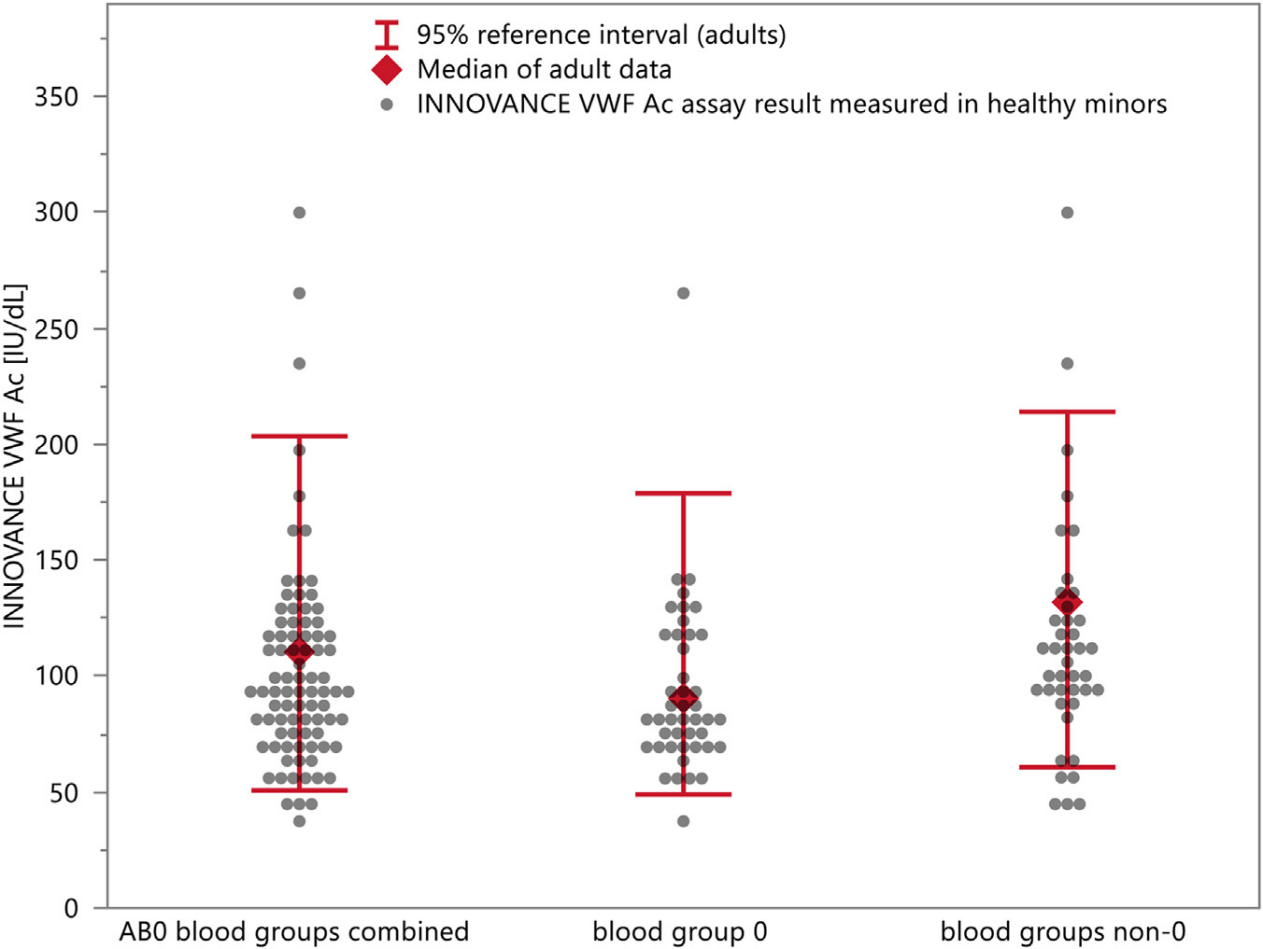
Comparison of the INNOVANCE VWF Ac assay measured in healthy minors with the reference range established for adults (≥18 years). The dots are the INNOVANCE VWF Ac assay values measured in healthy minors. The adult reference range is displayed with red whiskers (2.5th and 97.5th percentiles) and the red diamond (median). Supplementary Table S5 shows the results for the CS-2500 and CS-5100 analyzers and the BCS XP System in a table format, and Supplementary Table S6 shows the statistical comparison between adults and minors. The difference between adults and minors is significant in the parametric *t*-test and in the nonparametric Wilcoxon test for “blood groups non-O” and the entire dataset “ABO blood groups combined.” VWF, von Willebrand factor.

We included 85 healthy minors with race and ethnic diversity (<18 years of age) and measured their samples with the VWF:GPIbM assay on the BCS XP System and on the CS instruments (1 sample needed to be excluded from the CS-2500 analyzer study component because of insufficient volume). The population was balanced regarding gender and ABO blood group (*n* = 44 with blood group O and *n* = 41 with blood groups non-O). Twenty-five subjects (29%) were between 4 weeks and 2 years of age, 38 subjects (45%) were between 3 years and 12 years of age, and 12 subjects (22%) were between 13 and 17 years of age. The results in comparison with the reference ranges established for adults are depicted in Figure 4 and Supplementary Table S5. Most of the results (>85%) obtained for healthy minors were found within the respective reference ranges established for adults. Results outside the reference value were more often observed for subjects with non-O blood groups compared with those with blood group O. The statistical analysis demonstrated that values of minors were consistently lower than values for adult from both subgroups, “blood group O” and “blood groups non-O,” and, thus, also when evaluating both blood subgroups combined. Results in Supplementary Table S6 were calculated for log-transformed data and back-transformed. Differences and CIs are reported as ratios between geometric group means. For the analysis of the dataset “blood groups non-O” and the entire dataset (“ABO blood groups combined”), the difference is seen to be significant in both the parametric *t*-test and the nonparametric Wilcoxon test.

### 3.4 Reproducibility/precision

The VWF:GPIbM assay was demonstrated to be very precise over a range of VWF activity from 9 to 260 IU/dL, with coefficient of variations (CVs) mostly below 5%; the highest CV was 6.29% (Table 4). The reproducibility/precision observed on the CS-5100 and CS-2500 analyzers was similar (data shown in Supplementary Tables S7 and S8).

**Table 4:** Reproducibility/precision for the INNOVANCE VWF Ac assay on the BCS XP System.

Study	Plasma pools very low	Plasma pools low	Control P	Plasma pools medium	Control N	Plasma pools high	Plasma pools very high
Three sites, 5 days, 2 runs, 3 replicates (1 reagent/calibrator lot)							
Within-run	2.10	2.12	1.25	1.68	1.97	2.71	3.33
Between-run	1.84	1.02	1.12	0.70	1.05	2.29	1.18
Within-site	2.79	2.54	1.79	1.87	2.24	3.54	3.53
Between-site	2.08	5.58	4.98	2.13	1.92	5.20	3.91
Total (combined sites)	3.48	6.14	5.30	2.83	2.95	6.29	5.04
Mean of all measurements (*n* = 90)	9.1 IU/dL	22.8 IU/dL	29.0 IU/dL	49.3 IU/dL	87.8 IU/dL	128.3 IU/dL	251.6 IU/dL
Three reagent lots, 20 days, 2 runs, 2 replicates (1 site)							
Within-run	1.34	1.49	2.71	1.30	1.30	3.40	3.35
Between-run	1.20	3.12	1.54	0.62	0.62	1.75	2.98
Between-reagent lot	2.26	1.61	1.37	1.55	1.55	2.80	3.51
Total (combined sites)	2.90	3.82	3.40	2.23	2.23	5.20	6.02
Mean of all measurements (*n* = 240)	10.3 IU/dL	23.3 IU/dL	28.8 IU/dL	51.9 IU/dL	84.4 IU/dL	132.0 IU/dL	260.0 IU/dL

The table shows the coefficient of variation in percentage (%) for each precision characteristic of the study at *n* = 3 clinical sites (first line) and of the study in a Siemens laboratory with *n* = 3 reagent lots (second line).

## 4 Discussion

Deficiencies of the traditional VWF:RCo assay have spurred the development of multiple improvements. This work was undertaken to compare the performance of the INNOVANCE VWF Ac assay (an automated VWF:GPIbM assay) with other assays available in the United States, including an automated VWF:RCo assay, automated VWF:Ab assay, and a manual VWF:Ab assay. In addition to the method comparison studies, reference ranges were generated, and precision characteristics were evaluated.

The VWF:GPIbM assay correlated very well with platelet-binding activity measured by the automated VWF:RCo and the VWF:Ab assays (correlation coefficients, .919 and .971, respectively), with acceptable slopes and biases for both automated methods. Similarly, excellent correlations have been reported in the COMPASS-VWF (Comparison of assays to measure VWF activity) study [26] and in 3 large studies of previously characterized VWD cohorts [20,27,28]. Unlike other studies, this study also included a manual VWF:Ab assay. While the correlation coefficient for comparison between the VWF:GPIbM assay and the manual VWF:Ab assay was excellent (.97), the Passing–Bablok slope was 1.56 with the VWF:GPIbM assay, showing significant positive bias at 30 IU/dL and 50 IU/dL, but not at the intercept. The method comparison between the manual VWF:Ab assay and the other 2 methods (automated VWF:Ab and VWF:RCo assays) both showed a similar picture, with a high slope and high positive bias at 30 IU/dL and 50 IU/dL.

Despite the high overall comparability between the automated assays, disparities in single samples were observed. It is known that individual VWD patients may show wide discrepancies between VWF activity assays [28,29]. Investigating the reason for the observed discrepancies was beyond the scope of our study; data on the VWD genotype (including D1472H polymorphism) are not available.

The VWF:GPIbM method demonstrated tight correlations between the BCS XP and the CS analyzers, which allow laboratories to interchange platforms in clinical routine and pool data from different platforms for research purposes.

Reference ranges for the healthy adult population (not stratified for ABO blood group) demonstrated a 2.5th percentile level very close to the diagnostic cutoff for consideration of increased bleeding risk, as recently suggested in the ASH ISTH NHF WFH guideline on the diagnosis of VWD [1]. As previously observed [30], patients with blood group O showed a 2.5th percentile VWF level, which was 20% to 22% lower than that in the non-O blood group cohort. Appel et al. [31] have demonstrated that lower VWF levels are observed in a pediatric population >6 months compared with adults and that the effect is more pronounced in individuals with non-O blood group compared with those with O blood group. Consistent with this finding, as shown in Figure 4, the majority of pediatric samples had VWF:GPIbM activity levels below the median of the adult population (with significantly lower values for non-O blood groups, whereas statistical significance was not reached for blood group O), but only a minority of samples had levels below the 2.5th percentile of the adult reference range.

The reproducibility of the VWF:GPIbM assay was excellent over the entire range evaluated, from 9 to 260 IU/dL, consistent with the manufacturer’s suggested reportable range (4-300 IU/dL) for within-runs, between-runs, between-lots, and even between 3 clinical sites evaluated on the BCS XP analyzer. Similarly, excellent precision was demonstrated at the manufacturer’s facility for the CS-2500 and CS-5100 analyzers. High reproducibility is one of the major advantages of the automated VWF:GPIbM method compared with the VWF:RCo method, as was pointed out by James et al. [1].

The results of this study corroborate the initial observations of performance of the automated VWF:GPIbM assay [22] in a US population. They demonstrate linearity compared with other assays available in the United States and similar CV and comparability between VWF:GPIbM levels as reported by several automation platforms. Adult reference ranges observed in this current study are similar to those reported in 2014 on the BCS XP System [22], attesting to the stability of the assay over a decade.

The current study has several limitations. The VWF:GPIbM assay was compared only with a single formulation of the VWF:RCo assay. While the reportable range for the VWF:GPIbM assay has been validated to be as low as 4 IU/dL, comparisons were evaluated only within the manufacturer’s reportable range for the comparator VWF:RCo and VWF:Ab methods. There were few individuals included with prior diagnoses of type 2 VWD or alternative diagnoses such as AVWS and no patients with platelet-type VWD. The purpose of the current study was not to determine the accuracy of clinical diagnosis of VWD; the clinical diagnosis as recorded in the medical file was not questioned. Similarly, the VWF were not genotyped to determine the presence of pathogenic mutations or the presence of polymorphism D1472H, which may help to explain some of the observed disparities between VWF activity assays. The individual assays were each calibrated with the manufacturer’s recommended calibrator. The study did not include assays that are based on the ristocetin-induced binding of VWF to a recombinant GIPb fragment because none of these VWF:GPIbR assays are currently commercially available in the United States. Finally, *in vivo*, VWF is activated by shear stress, but all the assays incorporated in this study are performed in relatively static conditions. Also, we note that the reference interval studies did not include testing of VWF antigen; a correlation between VWF activity and VWF antigen in the normal population is thus not provided.

## 5 Conclusion

The VWF:GPIbM assay provides high precision and reports similar VWF platelet-binding activity as the automated VWF:RCo and VWF:Ab assays. The VWF:GPIbM assay demonstrates an expanded reportable range down to 4 IU/dL compared with the VWF:RCo assay and the automated VWF:Ab assay. Reference ranges determined with the VWF:GPIbM assay are consistent with those reported by other assays, and the correlations between results obtained on the BCS XP System and CS analyzers are remarkably consistent. These findings support the choice of the VWF:GPIbM assay for the clinical evaluation of patients with suspicion of VWD in compliance with the recommendation of the ASH ISTH NHF WFH guideline [1].
