# Supplementary material for: Evaluation of an automated von Willebrand factor glycoprotein IbM activity assay compared with 3 alternative von Willebrand factor activity assays
Source: Res Pract Thromb Haemost. 2024 Apr 26;8(4):102422. doi: 10.1016/j.rpth.2024.102422 (PMC11152683; doi:10.1016/j.rpth.2024.102422)
Supplement: Supplementary material [file mmc1.docx]

**SUPPLEMENTARY MATERIAL**

**Evaluation of an automated von Willebrand factor GPIbM activity assay compared to three alternative von Willebrand factor activity assays.**

Kenneth D. Friedman, Martina Böhm-Weigert, Nicole DeSimone, Dennis J. Dietzen, Charles Eby, Cynthia Flickinger, Walter Hoyer, Mareike Kahl, Kandice Kottke-Marchant, Thomas L. Ortel, Jürgen Patzke, Rhonda Porche-Sorbet, Steven W. Pipe, Morgan Stuart, Ayse Anil Timur and Ravindra Sarode

**SUPPLEMENTARY TABLE S1** VWF platelet-binding activity assays used in the study.

| **Assay** | **Manufacture** | **Nomenclature per VWF SSC of the ISTH** | **Measuring Interval**  **[IU/dL]** | **Calibrator** |
| --- | --- | --- | --- | --- |
| INNOVANCE VWF Ac | Siemens  Healthineers | VWF:GPIbM | 4 - 300 | Standard Human Plasma from Siemens Healthineers |
| BC von Willebrand Reagent | Siemens  Healthineers | VWF:RCo | 20 - 150 | Standard Human Plasma from Siemens Healthineers |
| HemosIL von Willebrand Factor Activity | Werfen | VWF:Ab | 19 - 390 | Calibration Plasma from Werfen |
| REAADS von Willebrand factor Activity test kit | Corgenix | VWF:Ab | 6.25 - 400 | Reference Plasma included in the test kit |

**SUPPLEMENTARY TABLE S2** Reasons for sample exclusion from method comparison studies between reagents.

| **Exclusion Criteria** | **VWF activity reagent (Number of samples)** |
| --- | --- |
| Quality control measurement not completed | BC von Willebrand Reagent on BCS XP (n=4) |
| Storage time exceeded | HemosIL VWF activity and REAADS VWF activity (n=17) |
| Diluted sample were not measured in Duke | HemosIL VWF activity and REAADS VWF activity (n=3) |
| Invalid test result | REAADS VWF activity (n=1) |
| Measurement failed | HemosIL VWF activity (n=1) |
| Sample hemolytic | HemosIL VWF activity and REAADS VWF activity (n=3) |

**SUPPLEMENTARY FIGURE S1** Bland-Altmann analysis for all reagent combinations.

The Bland-Altmann analysis compares two assays by plotting the difference between assay results against the average of the two assay results. The continuous red line depicts the mean difference between assays; dotted red lines depict 95% confidence limits for the mean difference. A: INNOVANCE VWF Ac assay compared with BC von Willebrand Reagent versus. B: INNOVANCE VWF Ac assay compared with HemosIL VWF Activity assay. C: INNOVANCE VWF Ac assay compared with REAADS VWF activity ELISA assay. D: BC Von Willebrand Reagent compared with HemosIL VWF activity assay. E: BC Von Willebrand Reagent with REAADS VWF activity ELISA assay. F: HemosIL VWF Activity assay compared with REAADS VWF activity ELISA assay. The BC von Willebrand Reagent and the INNOVANCE VWF Ac assay were performed on the BCS XP system; the HemosIL VWF Activity assay was performed on the ACL TOP system. The corresponding Passing-Bablok regression analysis are shown in Figure 2 of this publication.

mean difference: 23.3 IU/dL

**C**

mean difference: -3.6 IU/dL

**B**

mean difference: 7.9 IU/dL

**A**

mean difference: 26.9 IU/dL

**F**

**D**

mean difference: 15.4 IU/dL

**E**

mean difference: -11.5 IU/dL

**SUPPLEMENTARY TABLE S3** Samples identified as potential outlier in normal-quantile and outlier box plots analysis for at least one of the six method comparison between the VWF activity reagents.

| Sample Number | BC von Willebrand Reagent  [IU/dL] | INNOVANCE VWF Ac assay  [IU/dL] | REAADS VWF activity assay  [IU/dL] | HemosIL VWF activity assay  [IU/dL] |
| --- | --- | --- | --- | --- |
| 1 | 60.9 | 104.9 | 113.2 | 145.2 |
| 2 | >150 | 226.9 | 134.0 | 255.2 |
| 3 | 65.1 | 112.9 | 77.0 | 108.9 |
| 4 | >150 | 220.7 | 151.4 | 287.3 |
| 5 | 32.6 | 85.1 | 48.3 | 72.1 |

**SUPPLEMENTARY TABLE S4** Samples which reported results outside of the measuring interval with any of the four VWF activity reagents.

| Sample Number | BC von Willebrand Reagent  [IU/dL] | INNOVANCE VWF Ac assay  [IU/dL] | REAADS VWF activity assay  [IU/dL] | HemosIL VWF activity assay  [IU/dL] |
| --- | --- | --- | --- | --- |
| 1 | <20 | 20.6 | n/a* | n/a* |
| 2 | <20 | 10.9 | 19.2 | <19.0 |
| 3 | <20 | 26.4 | 13.4 | 21.4 |
| 4 | <20 | <4 | <6.25 | <19.0 |
| 5 | <20 | 4.9 | <6.25 | <19.0 |
| 6 | <20 | 15.9 | 21.9 | <19.0 |
| 7 | <20 | <20 / >25** | <6.25 | <19.0 |
| 8 | <20 | <4 | 6.6 | <19.0 |
| 9 | <20 | 15.9 | 11.1 | <19.0 |
| 10 | <20 | 23.7 | 21.8 | n/a* |
| 11 | <20 | 11.8 | 10.7 | <19.0 |
| 12 | <20 | <20 / >25** | 9.8 | <19.0 |
| 13 | <20 | 26.4 | n/a* | n/a* |
| 14 | <20 | <4 | <6.25 | <19.0 |
| 15 | <20 | 16.8 | n/a* | n/a* |
| 16 | <20 | <4 | <6.25 | <19.0 |
| 17 | <20 | 13.7 | 16.0 | <19.0 |
| 18 | <20 | 12.1 | 17.7 | 19.1 |
| 19 | <20 | 10.1 | 19.3 | 23.3 |
| 20 | <20 | <4 | 17.3 | 25.6 |
| 21 | <20 | 6.4 | 18.5 | 28.5 |
| 22 | <20 | 9.0 | <6.25 | <19.0 |
| 23 | <20 | 8.3 | <6.25 | <19.0 |
| 24 | <20 | 58.3 | 41.8 | 59.3 |
| 25 | <20 | <4 | <6.25 | <19.0 |
| 26 | <20 | 22.4 | <6.25 | <19.0 |
| 27 | 32.5 | 25.7 | 27.2 | <19.0 |
| 28 | >150 | >300 | n/a* | n/a* |
| 29 | >150 | 266.2 | n/a* | n/a* |
| 30 | >150 | 149.8 | n/a* | n/a* |
| 31 | >150 | 203.8 | 144.0 | 232.9 |
| 32 | >150 | 159.1 | 102.8 | 159.0 |
| 33 | >150 | 226.9 | 134.0 | 255.2 |
| 34 | >150 | >300 | 190.6 | >300.0 |
| 35 | >150 | 128.6 | 107.8 | 151.4 |
| 36 | >150 | 246.7 | 169.5 | 277.6 |
| 37 | >150 | 220.7 | 151.4 | 287.3 |
| 38 | n/a* | 11.2 | 17.5 | <19.0 |

*No result available because sample was excluded, or result was invalid

** The INNOVANCE VWF Ac assay result was flagged (<20 IU/dL in the normal setting and >25 IU/dL in the low setting)

**SUPPLEMENTARY TABLE S5** INNOVANCE VWF Ac assay results in healthy minors.

| **Analyzer** | **Number of donors** | **Minimum**  **[IU/dL]** | **Median**  **[IU/dL]** | **Maximum**  **[IU/dL]** | **Number of donors within RI adults (%)** | **Number of donors below / above RI adults** |
| --- | --- | --- | --- | --- | --- | --- |
| **BCS XP**  ABO blood groups combined  Blood Group O  Blood Group non-0 | 85  44  41 | 37.4  37.4  43.8 | 91.6  79.8  104.6 | >300.0  265.2  >300.0 | 78 (91.8)  42 (95.5)  34 (82.9) | 4 / 3  1 / 1  5 / 2 |
| **CS-2500**  ABO blood groups combined  Blood Group O  Blood Group non-O | 84  43  41 | 29.6  29.6  37.6 | 90.5  80.4  106.6 | >300.0  279.5  >300.0 | 76 (90.5)  41 (95.3)  36 (87.8) | 5 / 3  1 / 1  3 / 2 |
| **CS-5100**  ABO blood groups combined  Blood Group O  Blood Group non-0 | 85  44  41 | 29.0  29.0  37.5 | 88.0  77.6  106.1 | >300.0  239.8  >300.0 | 78 (91.8)  42 (95.5)  35 (85.4) | 4 / 3  1 / 1  4 / 2 |

VWF activity using the INNOVANCE VWF Ac assay was measured in 85 healthy minors (< 18 years). One sample could not be measured on the CS-2500 analyzer, because of insufficient volume. RI adults = Reference Interval established for healthy adults (≥ 18 years)

**SUPPLEMENTARY TABLE S6** Statistical comparison between the INNOVANCE VWF Ac assay results in healthy adults and healthy minors.

| **Group** | **Number of adults** | **Number of minors** | **Geometric Mean Ratio** | **Lower 95% confidence Limit for the ratio** | **Upper 95% confidence Limit for the ratio** | **p-value**  **t-test** | **p-value**  **Wilcoxon test** |
| --- | --- | --- | --- | --- | --- | --- | --- |
| **Blood Group O** | 150 | 44 | 0.931 | 0.828 | 1.046 | 0.2254 | 0.1944 |
| **Blood Groups non-O** | 152 | 41 | 0.823 | 0.716 | 0.946 | 0.0072 | 0.0009 |
| **ABO blood groups combined** | 302 | 85 | 0.872 | 0.794 | 0.956 | 0.0040 | 0.0011 |

The data were log-transformed and reported as ratio “healthy minor / adult” via back-transformation of the log-differences into ratios. For the parametric t-test confidence limits and p values are reported. The Wilcoxon p-value is additionally reported to confirm results of parametric t-test.

**SUPPLEMENTARY TABLE S7** Precision for the INNOVANCE VWF Ac assay on the CS-2500 system.

| **Study** | **Plasma pools**  **very low** | **Plasma pools**  **low** | **Control P** | **Plasma pools**  **medium** | **Control N** | **Plasma pools**  **high** | **Plasma pools**  **very high** |
| --- | --- | --- | --- | --- | --- | --- | --- |
| **20 days, 2 runs, 2 replicates**  **(One instrument/one reagent lot)**  Within-Run  Between-Run  Between-Day  Total (within site)  Mean of all measurements (n=80) | 2.20  0.00  0.99  2.41  8.1 IU/dL | 3.00  1.37  0.00  3.30  22.4 IU/dL | 2.38  1.13  0.00  2.63  28.8 IU/dL | 2.14  1.25  2.49  3.52  45.0 IU/dL | 3.03  1.39  0.00  3.33  82.6 IU/dL | 3.44  0.94  2.31  4.25  135.0 IU/dL | 2.60  1.61  0.00  3.06  272.1 IU/dL |
| **5 days, 2 runs, 4 replicates**  **(Three instruments/one reagent lot)**  Within-Run  Between-Run  Between-Day  Within-Instrument  Between-Instrument  Total (combined instruments)  Mean of all measurements (n=120) | 2.45  0.41  0.37  2.51  2.40  3.47  7.9 IU/dL | 5.01  0.09  0.00  5.09  3.27  6.05  21.8 IU/dL | 2.67  0.86  0.00  2.81  3.47  4.46  28.4 IU/dL | 2.19  1.54  1.41  3.02  2.74  4.08  45.1 IU/dL | 2.70  0.18  1.48  3.08  3.19  4.44  81.0 IU/dL | 3.19  0.88  0.49  3.34  4.56  5.65  131.5 IU/dL | 2.48  1.74  0.52  3.08  4.57  5.51  272.5 IU/dL |

The table shows the coefficient of variation (CV) in % for each precision characteristic evaluated at one site by measuring pools and controls on one instrument (first line) and on three instruments (second line).

**SUPPLEMENTARY TABLE S8** Precision for the INNOVANCE VWF Ac assay on the CS-5100 system.

| **Study** | **Plasma pools**  **very low** | **Plasma pools**  **low** | **Control P** | **Plasma pools**  **medium** | **Control N** | **Plasma pools**  **high** | **Plasma pools**  **very high** |
| --- | --- | --- | --- | --- | --- | --- | --- |
| **20 days, 2 runs, 2 replicates**  **(one instrument/one reagent lot)**  Within-Run  Between-Run  Between-Day  Total (within site)  Mean of all measurements (n=80) | 1.61  0.90  1.08  2.13  8.8 IU/dL | 2.08  1.32  0.00  2.44  23.4 IU/dL | 2.15  0.41  0.00  2.19  30.2 IU/dL | 1.24  1.36  2.10  2.79  46.4 IU/dL | 2.37  1.85  0.00  3.01  81.5 IU/dL | 1.44  1.55  2.86  3.56  134.0 IU/dL | 1.19  1.18  0.50  1.75  253.1 IU/dL |
| **5 days, 2 runs, 4 replicates**  **(three instruments/one reagent lot)**  Within-Run  Between-Run  Between-Day  Within-Instrument  Between-Instrument  Total (combined instruments)  Mean of all measurements (n=120) | 1.44  1.08  0.00  1.80  3.42  3.87  8.6 IU/dL | 2.65  0.82  0.62  2.84  1.80  3.36  23.3 IU/dL | 3.41  1.38  0.89  3.78  2.73  4.67  29.7 IU/dL | 1.13  1.75  0.39  2.12  2.71  3.44  45.3 IU/dL | 1.72  1.08  0.17  2.04  2.94  3.58  80.7 IU/dL | 1.32  1.09  0.09  1.72  3.06  3.51  133.1 IU/dL | 0.98  1.16  1.39  2.06  0.00  2.06  263.6 IU/dL |

The table shows the coefficient of variation (CV) in % for each precision characteristic evaluated at one site by measuring pools and controls on one instrument (first line) and on three instruments (second line).

The table shows the coefficient of variation (CV) in % for each precision characteristic evaluated at one site by measuring pools and controls on one instrument (first line) and on three instruments (second line).
